# An Address to the First Graduates of the Medical Department of the University of Michigan

**Published:** 1851-10

**Authors:** 


*An Address to the First Graduates of the Medical Department of the University of Michigan.* By Z. Pitcher, M. D., Regent of the University, and President of the State Medical Society.

We have read this address with great pleasure. It is what would be expected from an accomplished physician, and cultivated gentleman.

